# Clinical and psychological characteristics associated with negative beliefs and concerns about treatment necessity in rheumatic diseases

**DOI:** 10.1038/s41598-022-27046-5

**Published:** 2022-12-30

**Authors:** Sarah Tosato, Chiara Bonetto, Alice Zanini, Riccardo Bixio, Martina Marchel, Giulio Pacenza, Isotta Galvagni, Doriana Cristofalo, Elena Fracassi, Antonio Carletto

**Affiliations:** 1 Section of Psychiatry, Department of Neuroscience, Biomedicine and Movement Sciences, University of Verona, Section of Psychiatry, University of Verona, P.le Scuro 10, 37134 Verona, VR Italy; 2 Rheumatology Unit, Department of Medicine, University of Verona, Verona, Italy

**Keywords:** Psychology, Rheumatology

## Abstract

Identifying factors that influence problematic beliefs and behaviors related to pharmacotherapy may be useful for clinicians to improve the patients’ adherence. The study aims to assess patients’ beliefs about the necessity and concerns regarding pharmacotherapy in rheumatic diseases and attitude styles, and to investigate the association between clinical factors and negative beliefs about medication. A sample of 712 patients affected by Rheumatoid Arthritis, Psoriatic Arthritis, Ankylosing Spondylitis was enrolled. They were assessed using the Beliefs about Medicines Questionnaires-Specific (BMQ), the Simplified Disease Activity Index (SDAI), the Visual Analogue Scale for pain (VAS), the Chalder Fatigue Scale (CFQ) and the Health Assessment Questionnaire-Disability Index (HAQ-DI). The balance between benefits and costs in the BMQ-Specific was positive in the 79.4% of patients, negative in the 12.1% and equal in the 8.6%. SDAI, taking more than 5 medications, taking anti interleukin 6 (Anti-IL6) or biological disease-modifying antirheumatic drugs (bDMARDs), or targeted synthetic disease-modifying antirheumatic drugs (tsDMARDs), pain, and fatigue were significantly associated to higher Concerns. Having a longer disease duration was significantly associated with a higher Necessity, together with the current pharmacological treatments and the disability. The multivariate regression models estimated that higher pain and fatigue were associated to higher Concerns (*p* < 0.001), while a longer disease duration (*p* < 0.001) and all pharmacological treatments for a rheumatologic disease (*p* = 0.001) were associated to higher Necessity levels. A high length of disease, a low level of remission, a high number of total medications, the prescription of an Anti-IL6/bDMARDs/tsDMARDs drug, a high level of pain, fatigue and disability identified patients potentially less adherent to pharmacotherapy to be carefully looked after by clinicians.

## Introduction

Rheumatic diseases require often complex and long-term therapy in order to prevent exacerbations, control pain and improve the quality of life of patients. As in other chronic diseases^1^, it is extremely challenging to achieve compliance with pharmacological treatment in order to prevent progression of the disease and hospitalizations^2–4^. Compliance can be influenced by several factors as pain, disease duration, comorbidities, disability and individual beliefs about illness and medication^5–7^.

Beliefs about medication formed an attitude toward medication that can reflect doubts about personal need for treatment and concerns about potential adverse effects^8^. Several studies^1,9,10^ indicated that people who had stronger beliefs in the necessity of taking medicine were more inclined to adhere to the prescription. In contrast, those who were more concern about the side effects or the dependence liability were more likely to have intentional non-adherence. Thus, identifying clinical, psychological, personal factors that influence problematic beliefs and behaviors of the individual may be useful for the clinicians to improve the patients’ adherence.

In Rheumatoid Arthritis (RA), negative beliefs about medications are a predictor of non-compliance and other factors as younger age, female gender and level of education contribute to decrease adherence^11^. Indeed, clinical factors as number of medications, pain, fatigue, physical disability, duration of illness enhance the level of necessity and concerns beliefs^9,12^. Conversely, in Psoriatic Arthritis (PsA) or Ankylosing Spondylitis (AS), type of treatment, age, race or disease duration influenced beliefs about medicines and adherence to treatment^13^.

We investigated all these factors in a multi-diagnosis sample (RA, PsA and AS), in order to find the predictive factors of negative beliefs of medications that are common to all three diseases.

Specifically, the aim of this study was to assess patients’ beliefs about the necessity of treatment and concerns regarding pharmacotherapy in rheumatic diseases and to assess the attitude style to understand which are the doubts about personal need for treatment and concerns about potential adverse effects. The secondary aim is to investigate the association between clinical factors and negative beliefs about medication, in order to identify from the beginning the sociodemographic, clinical and psychological characteristics of patients that predict concerns to pharmacotherapy, according to the Necessity–Concerns Framework. The latter has been shown to explain non-adherence across a range of illnesses, including rheumatoid arthritis^1^. Our exploration can help clinicians to identify from the beginning those patients with negative beliefs about medication in order to implement preventive intervention in order to facilitate the assumption of therapy.

## Materials and methods

### Clinical sample

The sample consisted of a 1-year treated cohort of patients, aged 18 years or older, affected by Rheumatoid Arthritis (RA), according to the American College of Rheumatology (ACR)/European League Against Rheumatism (EULAR)^14^ or Psoriatic Arthritis (PsA), according to the CASPAR criterions^15^ or Ankylosing Spondylitis (AS) according to the Assessment of SpondyloArthritis international Society (ASAS) classification criteria for the axial/ peripheral spondyloarthritis^16^. In details, over the period of one year, all individuals in charge at the Unit of Rheumatology of the Verona University Hospital Trust (Italy) were asked to participate and to be assessed (see Pezzato et al. and Tosato et al.^17,18^ for details). All participants were already diagnosed and they were assessed once clinical stability was achieved, specifically if they did not change their prescribed pharmacotherapy in the last 3 months, in order to give his/her opinion about medications that had been used for quite some time. Patients were excluded if they were affected by fibromyalgia, connective tissue diseases (Systemic Lupus Erythematosus, Sjogren, sclerodermas, dermatomyositis, polymyositis), vasculitis, gout, infective arthritis, rheumatic polymyalgia or other severe systemic diseases.

The study was conducted in accordance with the Declaration of Helsinki^19^.

### Measurements

The assessments were performed in the framework of routine visit. A comprehensive set of standardized instruments was used to collect socio-demographic and clinical information.

Beliefs about medications were estimated by using the Beliefs about Medicines Questionnaires-Specific (BMQ)^20^: it consists of two subscales of five items each, measuring patients’ beliefs about the necessity of prescribed medication (Specific-Necessity) and their concerns about potential adverse consequences of taking the medication (Specific-Concern). Within the subscales, items are scored with a 5-point Likert scale, from 1 (strongly disagree) to 5 (strongly agree), and are summed to obtain a total score ranging from 5 to 25. Higher scores indicate stronger beliefs. By subtracting the concern score from the necessity score, a necessity–concerns differential score can be calculated (ranging from − 20 to + 20) where positive scores mean that patients perceive that benefits of medication outweigh costs^1^. For the present study, the Italian version of BMQ was used since it was demonstrated its good validity^21^.

As done previously^8^, necessity and concern scores were also dichotomized at the scale midpoint to create four attitudinal profiles: “skeptical” (low necessity, high concerns [score < 15 or ≥ 15, respectively]), “indifferent” (low necessity, low concerns), “ambivalent” (high necessity, high concerns), and “accepting” (high necessity, low concerns).

Several diseases characteristics were collected including: Simplified Disease Activity Index (SDAI)^22^, Visual Analogue Scale for pain (VAS)^23^, Chalder Fatigue Scale (CFQ)^24^ and Health Assessment Questionnaire-Disability Index (HAQ-DI)^25^.

The SDAI is scored by the numerical sum of: tender and swollen joint count (based on a 28‐joint assessment), patient and physician global assessment of disease activity (VAS 0–10 cm) and level of C‐reactive protein (mg/dl, normal < 1 mg/dl). The VAS is a unidimensional measure of pain intensity consisting of a 10 cm line, with two end points representing 0 (‘no pain’) and 10 (‘pain as bad as it could possibly be’). The CFQ is a self-administered questionnaire measuring the severity of physical and mental fatigue on two separate subscales. The HAQ-DI is a domain of the general HAQ (Health Assessment Questionnaire), a self-administered questionnaire. The HAQ-DI is assessed by the eight categories of dressing, arising, eating, walking, hygiene, reach, grip, and common activities and it is a good predictor of future disability costs.

Information about current and previous pharmacotherapy used for rheumatologic diseases were collected, categorizing it according to the following classification: “conventional synthetic disease-modifying antirheumatic drugs (“csDMARDs”); anti–tumor necrosis factor drugs (“Anti-TNF”); “csDMARDs plus anti-TNF” and “Other” including anti interleukin 6 (Anti-IL6) drugs, biological disease-modifying antirheumatic drugs (bDMARDs), targeted synthetic disease-modifying antirheumatic drugs (tsDMARDs) with or without csDMARDs. In details, in the clinical practice of the Unit of Rheumatology involved in the study, the first treatment of the patient was a csDMARD in monotherapy. In the event that the patient did not respond, an antiTNF was prescribed alone or in combination with csDMARDs. In the event that the patient did not respond again to this first-line therapy, the clinician prescribed an anti-IL6 drug or other bDMARDs (secukinunab, abatacept) or tsDMARDs (baricitinib, tofacitinib, upatacitinib) either in monotherapy or in combination with csDMARDs. The latter was the second-line therapy. The use of glucocorticoid and the number of medications taken for comorbidity were also collected.

### Statistical analysis

Categorical variables were described by frequencies and percentages; continuous variables were presented by means and standard deviations. Linear regression models estimated the association between each of the BMQ-Specific dimensions (dependent variable) and the patients’ characteristics (independent variables). A first set of models was estimated by entering all the independent variables belonging to each of the 4 conceptual blocks (socio-demographic characteristics, clinical characteristics, pharmacological treatments and subjective assessment of disease), separately for each block. After that, only those independent variables with *p* < 0.10 were entered in the final multivariate models, where Beta coefficients were considered significant at *p* < 0.05. Before applying the models, the assumption of Normality was checked by producing the frequency histograms. Analyses were performed by IBM Corp. Released 2021. IBM SPSS Statistics for Windows, Version 28.0. Armonk, NY: IBM Corp.

### Ethic approval and consent to participate

A written informed consent was obtained to all participants after receiving an accurate description of the study. The study was conducted in accordance with the Declaration of Helsinki and approved by the Ethics Committee of the Provinces of Verona and Rovigo (Ref. CESC15840, 2016).

## Results

The study sample was constituted by 712 patients (Table 1). The majority of them were females (70.2%), with a mean age of 57 years (SD 12), more than 70% were married, about 56% had a low educational level and about half sample was constituted by unemployed.

**Table 1:** Socio-demographic and Clinical characteristics of patients (n = 712).

Socio-demographic characteristics	Total sample (n = 712)
Age (years), mean (sd) Median (IQR)	57.2 (12.5) 58 (49–67)
Female, n (%)	500 (70.2%)
Low education, n (%)	397 (55.8%)
**Clinical characteristics**	
Diagnosis, n (%) Rheumatoid Arthritis Psoriatic Arthritis Ankylosing Spondylitis	435 (61.0%) 173 (24.2%) 104 (14.8%)
Disease duration (years), mean (sd) median (IQR)	11.6 (9.0) 10 (4–16)
Simple Disease Activity Index (SDAI), n (%) Remission (≤ 3.3) Low (3.3–11.0) Moderate (11.0–26.0) High (> 26.0)	58 (8.1%) 277 (38.9%) 332 (46.6%) 45 (6.4%)
Comorbidity, n (%)	638 (89.6%)
**Pharmacological treatments**	
Total number of medications (for all diseases), n (%) ≤ 5 > 5	451 (63.3%) 261 (36.7%)
Past number of medications for rheumatologic diseases, n (%) 0–1 2+	253 (35.5%) 459 (64.5%)
Pharmacological treatment, n (%) Only csDMARDs Only Anti-TNF csDMARDS plus anti-TNF Other (Anti-IL6/bDMARDs/tsDMARDS) with or without csDMARDs	197 (27.7%) 198 (27.8%) 160 (22.5%) 157 (22.0%)
Glucocorticoid treatment, n (%)	269 (37.8%)
**Subjective assessment of disease**	
Visual Analogue Scale for pain (VAS), mean (sd) (1 missing) median (IQR) 0 1 2 3 4 5 6 7 8 9 10	5.0 (2.6) 5 (3–7) 33 (4.6%) 57 (8.0%) 59 (8.3%) 75 (10.5%) 64 (9.0%) 100 (14.1%) 67 (9.4%) 112 (15.8%) 89 (12.5%9 40 (5.6%) 15 (2.1%)
Chalder Fatigue Scale (CFQ), mean (sd) median (IQR) 0–3 no fatigue 4 + fatigue	4.2 (4.2) 3 (0–7) 374 (52.5%) 338 (47.5%)
Health Assessment Questionnaire- Disability Index (HAQ-DI), mean (sd) median (IQR) ≤ 0.1 no disability 0.1–1 low disability 1–2 moderate disability > 2 high disability	0.7 (0.6) 0.6 (0.1–1.0) 221 (31.0%) 326 (45.8%) 140 (19.7%) 25 (3.5%)

Regarding clinical characteristics, 61% of patients were affected by rheumatoid arthritis, 24% by psoriatic arthritis and the remaining 15% by ankylosing spondylitis. The mean illness duration was 12 years (SD 9) and only 8% of patients was in remission. About 90% of the sample were affected by comorbidities. Thirty-seven percent of patients declared to take more than 5 medications. The past treatment specific for a rheumatologic disease was constituted by at least 2 medications for 64.5% of patients; the current treatment was constituted by csDMARDs (27.7%), Anti-TNF (27.8%), their combination (22.5%) and Anti-IL6/bDMARDs/tsDMARDs with or without csDMARDS (22.0%). The glucocorticoid treatment was taken by 269 patients (37.8%).

The mean VAS score ranged from ‘8’ to ‘10’ (upper part of the scale indicating high pain levels) in the 20.2% of sample, while in the CFQ the 47.5% of patients scored above the cutoff. Finally, for the HAQ-DI, the 23.2% of patients indicated at least a moderate disability (HAQ-DI > 1).

### Beliefs

Considering the BMQ-Specific dimensions, the mean scores were 20.2 (SD 2.7) for the Necessity and 16.5 (SD 3.3) for the Concerns (Table 2). In detail, for the Necessity dimension the highest percentages of agreement were reported for the items ‘My medicines protect me from becoming worse’ (89.9%) and ‘Without any medicines I would become very ill’ (85.1%), while for the Concerns dimension the two items with the highest percentages were ‘I sometimes worry about the long-term effects of my medicines’ (69.5%) and ‘I sometimes worry about becoming too dependent on my medicines’ (55.1%).

**Table 2:** Beliefs about Medicines Questionnaire (BMQ)-Specific dimensions (n = 712).

BMQ-Specific dimensions	N (%) agreeing or strongly agreeing
Necessity, mean (sd) Median (IQR) My health, at present, depends on my medicines My life would be impossible without my medicines Without my medicines I would become very ill My health in the future will depend on my medicines My medicines protect me from becoming worse	20.2 (2.7) 20 (19–22) 561 (78.8%) 561 (78.8%) 606 (85.1%) 553 (77.7%) 640 (89.9%)
Concerns, mean (sd) Median (IQR) Having to take medicines worries me I sometimes worry about the long-term effects of my medicines My medicines are a mystery to me My medicines disrupt my life I sometimes worry about becoming too dependent on my medicines	16.5 (3.3) 17 (14–19) 364 (51.1%) 495 (69.5%) 220 (30.9%) 280 (39.3%) 392 (55.1%)
**BMQ-Specific Necessity-Concerns differential**	**N (%)**
Necessity-Concerns, mean (sd) Median (IQR) Necessity > Concerns Necessity = Concerns Necessity < Concerns	3.7 (4.1) 3 (1–6) 565 (79.4%) 61 (8.6%) 86 (12.1%)
BMQ-Specific attitudinal groups*	**N (%)**
Skeptical (low Necessity, high Concerns) Indifferent (low Necessity, low Concerns) Ambivalent (high Necessity, high Concerns) Accepting (high Necessity, low Concerns)	14 (2.0%) 13 (1.8%) 451 (63.3%) 234 (32.9%)

*Necessity and Concerns dimensions were divided at the scale midpoint 15 (low Concerns ≤ 15, high Concerns > 15; low Necessity ≤ 15, high Necessity > 15).

In the cost–benefit analysis, the majority of patients (79.4%) declared that benefits exceeded costs, 12.1% that costs overcame benefits and the remaining 8.6% reached a balance between benefits and costs.

When considering the four attitude groups defined by Necessity and Concerns levels, the ambivalent group (low Necessity, high Concerns) was the most frequent (63.3%), followed by the accepting group (high Necessity, low Concerns) (32.9%). Skeptical and indifferent patients constituted residual groups of 2% and 1.8%, respectively.

### Association between patients’ characteristics and beliefs

The Beta coefficients (*p* values) of the association between the BMQ-Specific dimensions and the characteristics belonging to each a-priori block are given in Table 3.

**Table 3:** Linear regression models for BMQ-Specific dimensions: Beta coefficients (*p* value) within each block (n = 712).

	BMQ-Specific Beta coefficients (*p* value) within each block	
Independent variables	Concerns	Necessity
**Block 1: socio-demographic characteristics**	**Adj-R**^**2**^** = 0.9%**	**Adj-R**^**2**^** = 0.4%**
Age (years)	0.02 (0.151)	0.01 (0.114)
Female	0.37 (0.179)	− 0.40 (0.070)
Low education	0.43 (0.111)	− 0.04 (0.869)
**Block 2: clinical characteristics**	**Adj-R**^**2**^** = 3.1%**	**Adj-R**^**2**^** = 3.7%**
Diagnosis (Ref. Rheumatoid Arthritis) Psoriatic Arthritis Ankylosing Spondylitis	− 0.54 (0.071) − 0.44 (0.230)	0.24 (0.316) − 0.17 (0.561)
Disease duration (years)	0.01 (0.898)	0.06 (< 0.001)
SDAI (Ref. Remission) Low Moderate High	1.69 (< 0.001) 2.17 (< 0.001) 2.64 (< 0.001)	0.23 (0.553) 0.09 (0.801) − 0.55 (0.294)
Comorbidity	0.01 (0.974)	0.31 (0.347)
**Block 3: pharmacological treatments**	**Adj-R**^**2**^** = 1.8%**	**Adj-R**^**2**^** = 4.7%**
Total number of medications (for all diseases) (Ref. ≤ 5) > 5	0.56 (0.045)	0.31 (0.154)
Past number of medications for rheumatological diseases (Ref. 0–1) 2+	− 0.10 (0.725)	0.20 (0.357)
Pharmacological treatment (Ref. csDMARDS) Anti-TNF csDMARDS plus anti-TNF Other with or without csDMARDS	− 0.03 (0.940) 0.06 (0.875) 0.87 (0.024)	0.89 (0.001) 1.48 (< 0.001) 1.18 (< 0.001)
Glucocorticoid treatment	0.34 (0.218)	0.07 (0.761)
**Block 4: subjective assessment of disease**	**Adj-R**^**2**^** = 6.6%**	**Adj-R**^**2**^** = 0.9%**
VAS Scale	0.20 (< 0.001)	− 0.05 (0.254)
CFQ total score	0.12 (< 0.001)	0.01 (0.891)
HAQ total score	0.02 (0.945)	0.54 (0.004)

By considering the Concern scale (SC), the level of disease activity index (SDAI) was significantly (*p* < 0.001) associated, with a dose–response trend (Beta coefficients for each level: low 1.69, moderate 2.17, high 2.64). In addition, taking more than 5 medications was associated with higher SC levels (Beta 0.56, *p* value 0.045). The category “Anti-IL6/bDMARDS/tsDMARDS with or without csDMARDs” was positively associated to SC score (Beta 0.87, *p* value 0.024). Regarding the subjective assessment of disease, higher pain (VAS) and fatigue (CFQ) were associated to higher SC levels (Beta 0.20, *p* value < 0.001 and Beta 0.12, *p* value < 0.001, respectively).

By considering the Necessity scale (SN), being a female gave an indication of lower SN levels (Beta − 0.40, *p* value 0.070). Regarding clinical characteristics, only having a longer disease duration was associated with a higher SN (Beta 0.06, *p* value < 0.001). All the current pharmacological treatments for the rheumatologic disease were positively associated to higher SN levels with respect to the csDMARDs (Beta 0.89, 1.48 and 1.18, respectively; *p* value 0.001). In the subjective assessment of disease block, only the disability (HAQ-DI) was associated to SN (Beta 0.54, *p* value 0.004).

The multivariate regression models showed that only higher pain and fatigue indexes were associated to higher Concerns (Beta 0.15 and 0.12, respectively; *p* value < 0.001), while a longer disease duration (Beta 0.04, *p* value < 0.001) and all pharmacological treatments for a rheumatologic disease (Beta with respect to csDMARDS: 0.84, 1.31 and 1.07, respectively; *p* value 0.001) were associated to higher Necessity levels (Table 4).

**Table 4:** Multivariate linear regression models for BMQ-Specific dimensions: Beta coefficients (*p* value) (n = 712) [Only variables significant at *p* < 0.10 in the within-block regression models entered the multivariate final model].

Independent variables	BMQ-specific Beta coefficients (*p* value)	
	Concerns	Necessity
**Female**		− 0.40 (0.070)
**Disease duration (years)**		0.04 (< 0.001)
**SDAI (Ref. Remission)**		
Low	0.94 (0.056)	
Moderate	0.78 (0.150)	
High	0.98 (0.183)	
**Total number of medications (for all diseases) (Ref ≤ 5)**		
> 5	0.33 (0.208)	
**Pharmacological treatment (Ref. csDMARDs)**		
Anti-TNF	− 0.08 (0.794)	0.84 (0.001)
csDMARDs plus anti-TNF	0.13 (0.714)	1.31 (< 0.001)
Other with or without csDMARDs	0.61 (0.082)	1.07 (< 0.001)
**VAS Scale**	0.15 (0.015)	
**CFQ total score**	0.12 (< 0.001)	
**HAQ total score**		0.26 (0.099)
**F (df1, df2), *p* value**	**F (9, 701) = 7.23, *p* < 0.001**	**F (6, 705) = 10.44, *p* < 0.001**
**Adj-R**^**2**^	**7.3%**	**7.4%**

## Discussion

To our knowledge, this is the first study testing both the beliefs and attitude towards medications not only in a sample of patients affected by RA but also in PsA and AS.

The main finding is that, according to the Necessity-Concerns Framework^9^, patients showed higher Necessity score than Concern score and more than three-quarters of them had positive beliefs about the necessity of their medication. This result indicated a sample that believed in the necessity of their medication for maintaining health, and it is confirmed by the long history of therapies taken by our patients that may result in feelings of dependency on their therapy^26^. However, almost half the participants also expressed strong concerns about potential adverse effects and addiction.

The presence of higher necessity than concern beliefs seemed in line with previous findings found in sample of RA patients^9,27–29^ showing that a long history of disease may have led to the development of the beliefs of necessity treatments together with the concerns about side-effects. More interestingly, the results of the present study extended the scarce evidence in Psoriatic Arthritis and Ankylosing Spondylitis, where the only study was conducted in patients with a shorter history of disease than our^30^.

In addition, the sample was constituted by subjects with mainly Ambivalent attitude, with high necessity and high concern’s beliefs about medicines. It was found that, while the necessity beliefs were about importance for the maintenance of current health and for future health (“My medicines protect me from becoming worse”, “Without my medicines I would become very ill”), concern beliefs were about the negative long-term effects and to become dependent upon medications (“I sometimes worry about the long-term effects of my medicines” “I sometimes worry about becoming too dependent on my medicines”). This attitude is not so useful since it was a powerful predictor of less adherence to treatments^31^. More worthwhile for the patients is to have an acceptance attitude (high necessity, low concerns)^31–33^. Therefore, part of the rheumatologist's time should be devoted to improving the patient's attitude towards medications, managing specifically concerns related to pharmacotherapy, by investigating in an active way in each patient above all what his/her concerns are and providing timely and in-depth answers. This work could change the attitude of patients from ambivalent to acceptance one, ensuring a more stable adherence to treatment.

About the sociodemographic factors, it was found that beliefs about medication in rheumatic disease were not be influenced by these, maybe because the main characteristics of disease are the relevant factors that influence the patient’s beliefs, independently. This result extended previous findings in RA^9^ in a larger and younger sample affected also by SpA and AS. Specifically, it was found that age, gender and level of education showed no association with medication beliefs.

So, about the clinical factors, disease duration and level of remission were found associated with necessity and concerns beliefs. A higher length of disease increased necessity beliefs, consistently with the patient’s conviction of the importance to take their medication to manage the disease^9,12^. Lower level of remission increased concern beliefs, suggesting that patients with higher disease burden were more concerned about potential side effects and dependency. In previous studies^13,34^, these findings were limited on the use of specific class of medications (i.e. anti-TNF, csDMARDs or the combination of two), while in the present study it was investigated beliefs on all classes of medications that the patient can take and also on Anti-IL6/bDMARDs/tsDMARDs.

About the pharmacological treatment, taking more than 5 medications for comorbidities and the class of rheumatological treatment, especially “Anti-IL6/bDMARDs/tsDMARDs”, increased necessity and concern beliefs. It was found that as the number of medications taken increases, concerns beliefs increase. Previous studies^7,35,36^ were focused on the correlation between beliefs about medication and rheumatic pharmacotherapy while the present study is the first one investigating also the beliefs about medications related to all comorbidities that affected patients. It was found that the class of rheumatological treatment had a negative effect on beliefs: specifically, the class “other pharmacotherapy with or without csDMARDs” is the one that increased necessity and concern beliefs. This type of treatment is prescribed when the previous options have not been effective on symptoms of rheumatic disease. As previously reported^35^, patients taking this medication expressed the view that confidence on the effectiveness of the treatment decreased, because they had taken several medications in the past without achieving the remission. The fact that they had experienced therapeutic failures or adverse effects may have increased their distrust.

Finally, also greater pain, fatigue and level of disability increased beliefs about necessity and concern. As previously reported^9,12^, level of pain and fatigue increased concerns beliefs, because medication seems not work completely in the managing of characteristics of the disease that impact negatively on the quality of life and functioning. The level of disability has increased beliefs about necessity, probably because the patients’ belief that without medication even the smallest daily activities become complicated.

When discussed these results we should take in account several strengths and limitations. One of the strength is that our study recruited a cohort of stable patients who have not changed their prescribed pharmacotherapy in the last 3 months. Consequently, the beliefs of necessity and concerns about treatment rifer to medications that patients for quite long time and presumably did not it does not depend by an acute episode of diseases. Secondly, the patients are in charge at a Unit of Rheumatology and received regular follow-up visit, thus, since it is assessed in a real world setting it can be assumed that the beliefs are representative of those of patients suffering from rheumatological diseases. Thirdly, while previously beliefs about medicines were investigated only in relationship with the class and the total of number of medications only for rheumatic disease, in the present study the treatments for comorbidities were also be taken in account. This allowed to test with as much accuracy as possible the concern and necessity beliefs about medications in general, considering the high prevalence of comorbidities in rheumatic diseases.

This study was subjected to several limitations. First, we did not assess the level of adherence to pharmacotherapy through specific questionnaires as the Compliance Questionnaire Rheumatology (CQR)^37^ or the Medication Adherence Report Scale (MARS)^38^. Since the present study is a naturalist cohort, the information about adherence was obtained through a patient’s self-report to physician. In this context, it should be highlighted that it is consolidated practice of the Unit of Rheumatology to ask the patient if he/she takes the prescribed medications, if he/she forgets to take the pills at each visit.

These results suggest that beliefs about medication and several factors, which in turn influence medication adherence and uptake, may reflect the general concerns and mistrust of pharmacotherapeutic agents. We have defined a profile of patient potentially less adherent to pharmacotherapy characterized by high length of disease, low level of remission, high number of total medications taken, the class of medications taken, high level of pain, fatigue and disability. Thus, clinicians had to pay attention at a patient presenting with these characteristics, because he/she could be more vulnerable to become patient non-adherent to treatment in long time.

## Conclusions

In conclusion, clinicians should develop a strategic approach to facilitate adherence, using a psychoeducational approach to actively explore concerns about medications, to give timely answers to questions about adverse effects, more focused information on treatment, in order to prevent high level of negative beliefs about medication and, in consequences, less adherence to treatment.

## Data Availability

The data that support the findings of the article are not publicly available but can be provided by the corresponding author (ST) on reasonable request.

## Abbreviations

RA: Rheumatoid Arthritis
PsA: Psoriatic Arthritis
AS: Ankylosing Spondylitis
BMQ: Beliefs about Medicines Questionnaires—Specific
SDAI: Simplified Disease Activity Index
VAS: Visual Analogue Scale
CFQ: Chalder Fatigue Questionnaire
HAQ-DI: Health Assessment Questionnaire—Disability Index
csDMARDs: Conventional Synthetic Disease Modifying Antirheumatic Drugs
Anti-TNF: Anti–tumor necrosis factor
Anti-Il6: Anti-interleukin-6
bDMARDs: Biological disease-modifying antirheumatic drugs
tsDMARDS: Targeted synthetic Disease Modifying Antirheumatic Drugs
SC: Concern scale
SN: Necessity scale
NCF: Necessity—concern framework
MARS: Medication Adherence Report Scale
